# Effects of palmatine on BDNF/TrkB-mediated trigeminal neuralgia

**DOI:** 10.1038/s41598-020-61969-1

**Published:** 2020-03-19

**Authors:** Lijuan Liu, Lingkun He, Cancan Yin, Ruoyu Huang, Wenhao Shen, Huixiang Ge, Mengyun Sun, Shujuan Li, Yun Gao, Wei Xiong

**Affiliations:** 1 Affiliated Stomatological Hospital of Nanchang University, Nanchang, Jiangxi China; 2 First Affiliated Hospital of Nanchang University, Nanchang, Jiangxi China; 3 Department of Physiology, Basic Medical College, Nanchang University, Nanchang, Jiangxi China; 4 Second Clinic Medical College of Grade 2017, Nanchang University, Nanchang, Jiangxi China; 5 Jiangxi Provincial Key Laboratory of Oral Biomedicine, Nanchang, Jiangxi China

**Keywords:** Somatic system, Neuropathic pain

## Abstract

Trigeminal neuralgia (TN), a sudden, needle-like pain in the distribution area of the trigeminal nerve, can seriously affect the physical and mental health of patients. In chronic pain conditions including TN, increased levels of brain-derived neurotrophic factor (BDNF) may enhance pain transmission. This study compares the effect of palmatine administration on the expression of BDNF and its receptor TrkB (tropomyosin receptor kinase B) in trigeminal ganglion cells of Sprague-Dawley rats in a sham versus TN model group. Within 14 days of surgery, the mechanical allodynia threshold of the TN group was significantly lower than that of the sham group, while the TN + palmatine group had a higher mechanical pain sensitivity threshold than the TN group (p < 0.05). Real-time quantitative PCR, immunohistochemistry, and immunofluorescence showed that BDNF and TrkB expression in the TN group was higher than that in the sham group, while palmatine treatment could reverse these changes. Western blotting showed that palmatine treatment could reduce the elevated phosphorylation of extracellular signal-regulated kinases 1/2 (ERK1/2) in TN rats. Thus, the BDNF/TrkB pathway may be involved in the pain transmission process of TN, and palmatine treatment may reduce pain transmission by inhibiting the BDNF/TrkB pathway and suppressing ERK1/2 phosphorylation.

## Introduction

Trigeminal neuralgia (TN) is a sharp, sudden pain in the trigeminal nerve region, the largest pair of nerves in the brain. TN causes intractable facial pain, which can seriously affect the patient’s quality of life^1^. Currently, treating TN is challenging. Pharmacological treatments, including carbamazepine, oxcarbazepine, and other anticonvulsants and antipsychotics, have achieved varying degrees of success. However, their side effects and potential loss in efficacy over time has prompted the search for other drugs that can achieve more comprehensive and long-lasting pain control^2^. Furthermore, carbamazepine and oxcarbazepine confer a significant risk of side effects and complications^3^. Indeed, both are prohibited to primary TN patients with an atrioventricular block^4^. Moreover long-term use of these medications can cause significant side effects such as drowsiness, rashes, and tremors^5^. Some TN patients may end up needing surgery, particularly if pharmacotherapy is ineffective or they become resistant to the drugs. Surgical treatments include microvascular decompression, gamma knife radio surgery treatment (GKRS), stereotactic radio surgery, and other invasive approaches^6–8^. Unfortunately, invasive treatments for TN can cause severe, diverse, and unpredictable side effects. For example, GKRS treatment for TN has a 50% chance of failure or recurrence, and the expensive surgery increases psychological and living burden of patients^9^. Thus, we need to further understand the molecular mechanism of TN to find new drug targets for the prevention and treatment of this debilitating disease.

Brain-derived neurotrophic factor (BDNF) is an important molecule for neurogenesis and neuronal survival^10,11^. Neuronal activity induces the synthesis and release of BDNF, which then recruits and activates cohesive proteins involved in various cellular signalling pathways^12^. Many studies have confirmed that BDNF and its tropomyosin receptor kinase B (TrkB) are associated with chronic pain transmission^13^. Recently, after repeated dural stimulation for trigeminal allodynia, some scholars discovered an increased expression of BDNF and TrkB in the trigeminal nucleus caudalis^14,15^. Hence, we speculate that the BDNF-TrkB signalling may be involved in the development of TN.

Palmatine is an alkaloid derived from dried rhizomes, a Chinese plant, and has been used to treat infectious diseases in China for thousands of years^16,17^. Palmatine has many therapeutic effects, has long-term presence in the bloodstream, and penetrates the blood-brain barrier^18,19^. For instance, palmatine may inhibit growth and the invasion of cancer, suppress inflammatory cytokines, and alleviate comorbid diabetic neuropathic pain and depression^20–22^. The study aimed to investigate whether palmatine treatment can affect the expression of BDNF/TrkB in the trigeminal ganglion (TG) of TN model rats and its possible molecular mechanism, which may provide new insights into the prevention and treatment of TN.

## Results

### Effects of palmatine on trigeminal neuropathic mechanical allodynia

**Figure 1:**
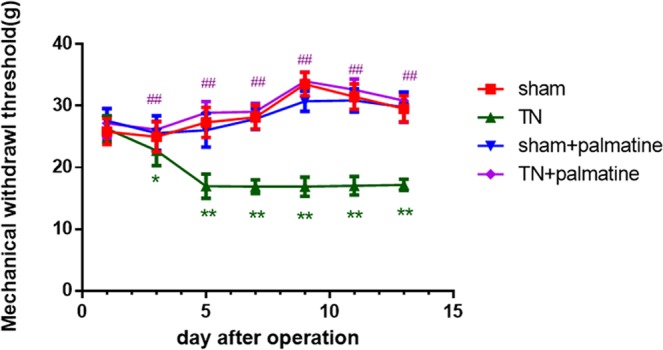
Mechanical withdrawal threshold (MWT). Three to fourteen days after the operation, the pain threshold in the TN group significantly decreased compared with the shams. Palmatine treatment noticeably reduced pain sensitivity of TN rats. Data are presented as mean ± SD, n = 12. *p < 0.05 *vs* sham group; **p < 0.01 *vs* sham group; ^##^p < 0.01 *vs* TN group.

The pain threshold in the TN group was significantly decreased in comparison with that in the sham group from the 3rd to 14th day after operation [d1: p = 0.128, F (3,44) = 1.996; d3: p < 0.05, F (3,44) = 4.945; d5: p < 0.01, F (3.44) = 66.551; d7: p < 0.01, F (3.44) = 164.307; d9: p < 0.01, F (3.44) = 193.993; d11: p < 0.01, F (3.44) =198.512; d13: p < 0.01, F (3.44) = 152.246. ANOVA was used]. We found that palmatine treatment noticeably reduced the pain sensitivity of TN rats for 2 weeks. See Fig. 1.

### Palmatine reduced the expression levels of BDNF/TrkB in the TG of TN rats

**Figure 2:**
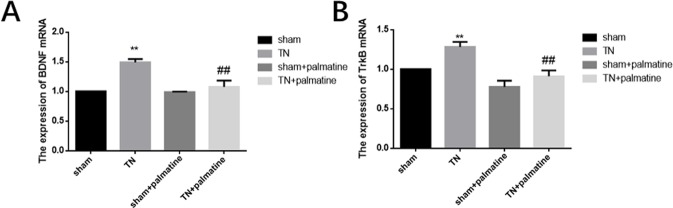
qPCR analysis. BDNF(**A**) and TrkB(**B**) mRNA levels in the TG of TN rats were higher than the shams. Palmatine treatment noticeably decreased these changes. Data are presented as mean ± SD, n = 6. **p < 0.01 vs sham group; ^##^p < 0.01 *vs* TN group.

Through qPCR analysis, we detected the mRNA expression of BDNF/TrkB in the TG of each group. These results showed that the expression of BDNF and TrkB mRNA in the TG of TN rats was higher than those in the sham group. The expression of BDNF and TrkB mRNA in TN + palmatine group was significantly decreased by palmatine treatment compared to the TN group. [BDNF: p < 0.01, F (3,20) = 49.115; TrkB: p < 0.01, F (3,20) = 35.660] see Fig. 2.

**Figure 3:**
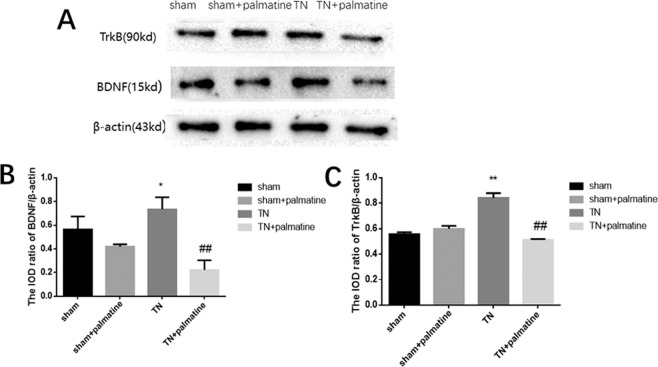
Western blot analysis. BDNF(**A**) and TrkB(**B**) protein levels in the TG of TN rats were higher than the shams. Palmatine treatment noticeably reversed these changes. Data are presented as mean ± SD, n = 6. **p < 0.01 *vs* sham group; ^##^p < 0.01 *vs* TN group.

Western blotting was used to measure the levels of BDNF/TrkB protein in the TG. The results showed that the protein expression of BDNF and TrkB in the TN group was higher than that in the sham group. The protein expression of BDNF/TrkB in the TN + palmatine group was lower than the TN group [BDNF: p < 0.01, F (3,20) =19.171; TrkB: p < 0.01, F (3,20) =113.892; see Fig. 3].

**Figure 4:**
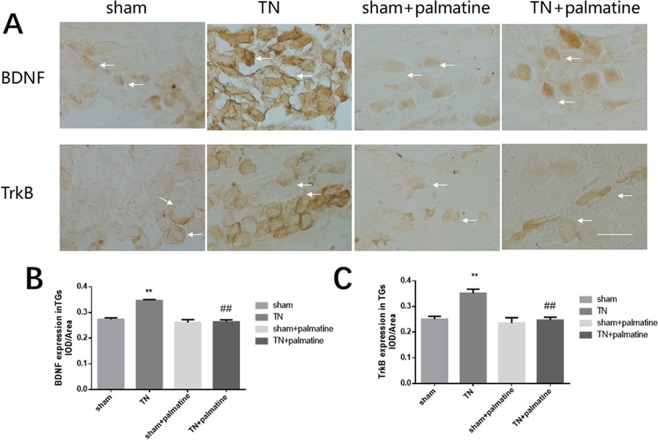
Immunohistochemistry analysis. The integrated optical density (IOD)/Area of BDNF and TrkB staining in the TG of TN rats were higher than the shams. Palmatine treatment noticeably reduced these changes. Arrows indicate immunostained cells. Scale bar: 100 μm. Data are presented as mean ± SD, n = 6. **p < 0.01 *vs* sham group; ^##^p < 0.01 *vs* TN group.

Immunohistochemistry was used to detect BDNF and TrkB immunoreactivities in the TG. The results showed that the expression of BDNF/TrkB receptor in the TG of TN group rats was higher than that in the sham rats, but palmatine treatment could reduce this change [BDNF: p < 0.01, F (3,20) = 87.760; TrkB: p < 0.01, F (3,20) = 37.211] (see Fig. 4).

**Figure 5:**
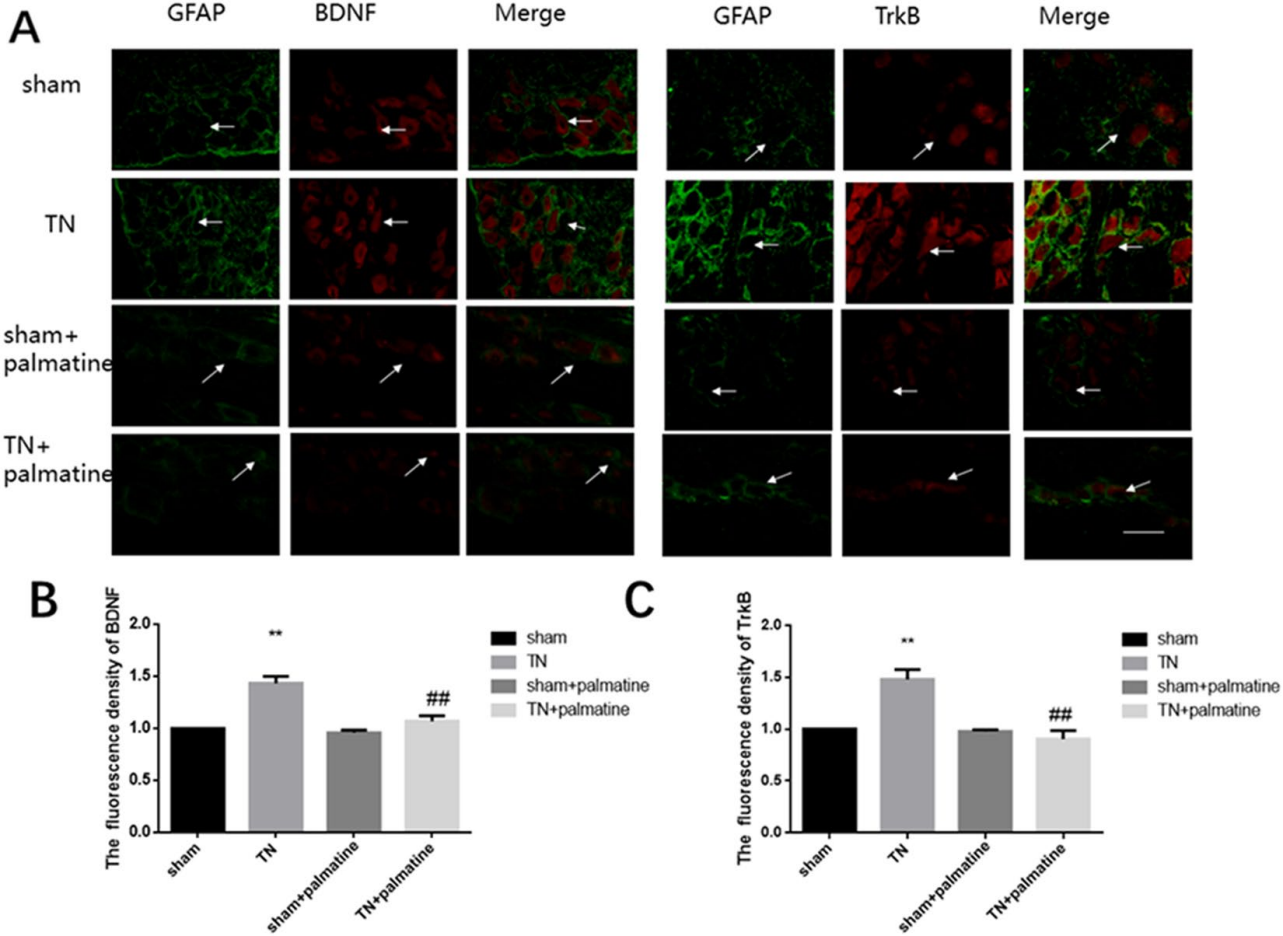
Immunofluorescences analysis. BDNF or TrkB receptor expression were shown in the TG. There was little GFAP co-expression with BDNF or TrkB receptor. Arrows indicate neuroglia and neurons emitting green and red fluorescence, respectively. Scale bar: 100 μm. Data are presented as mean ± SD, n = 6. **p < 0.01 *vs* sham group; ^##^p < 0.01 *vs* TN group.

Using immunofluorescence we also detected the immunoreactivities of the BDNF/TrkB. The co-expression of the BDNF or TrkB receptor and glial fibrillary acidic portein (GFAP) in the 4 groups on the 14th day is shown in Fig. 5. Because there was little co-expression of GFAP with either BDNF or TrkB receptor, we confirmed that the BDNF and TrkB receptor were expressed mainly in TG neurons [BDNF: p < 0.01, F (3,20) = 72.024; TrkB: p < 0.05, F (3,20) = 51.027] (see Fig. 5).

These results indicate that palmatine treatment can reduce the levels of BDNF/TrkB in the TG of TN rats.

### Effects of palmatine on the ERK1/2 and phosphorylation of ERK1/2 in the TG of TN rats

**Figure 6:**
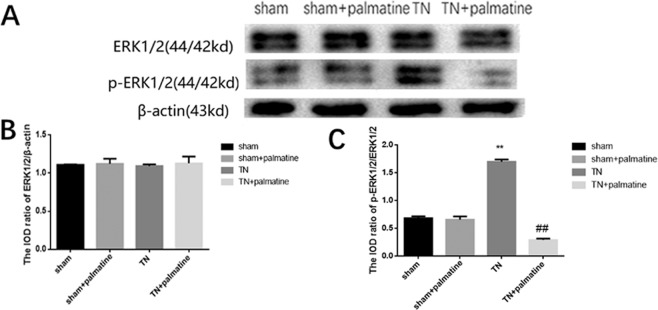
Western blot analysis. Integrated optical density (IOD) ratio of ERK1/2 to β-actin showed no significant differences between the groups. The IOD ratio of p-ERK1/2 to ERK1/2 in the TN group was higher than the shams. Palmatine treatment significantly reduced this phosphorylation. Data are presented as mean ± SD, n = 6. **p < 0.01 *vs* sham group; ^##^p < 0.01 *vs* TN group.

Through a Western blot analysis, we detected ERK1/2 and its phosphorylation in the TG. The integrated optical density (IOD)/Area ratio of ERK1/2 to β-actin showed no significant differences in the four groups (p > 0.05). Meanwhile, the IOD/Area ratio of p-ERK1/2 to ERK1/2 in the TN group were higher than in the sham group, but the IOD/Area ratio of p-ERK1/2 to ERK1/2 in the TN + palmatine group was significantly reduced compared to the TN group [ERK/β-actin: p > 0.05, F (3,20) = 0.375; p-ERK/ERK: p < 0.01, F (3,20) = 684.695] (See Fig. 6).

## Discussion

Although TN, a severe facial pain disorder, has been studied for decades, neither medicines nor surgical treatments can reach satisfactory effects^23^. The molecular mechanism of TN remains unclear and needs further investigation. The TG contains the primary sensory neurons of the head and face for pain transmission. Thus, the involved molecules in the TG have become a hot topic in TN research.

BDNF is a neurotrophin involved in the growth, differentiation and apoptosis of neurons^24^. Current research confirms that BDNF is related to pain. For instance, BDNF has been shown to be involved in the pathogenesis of cancer-induced pain, diabetic neuropathic pain and inflammatory pain^25–27^. However, reports on the effects of BDNF on TN are currently rare^28^; therefore, further studies on their role in TN would be useful.

In our experiment, we found that the TN group had an increased BDNF/TrkB expression in the TG. Thus, we presume that this increase of BDNF/TrkB may have promoted TN and may be involved in the sensitization process. Furthermore, our immunofluorescence results showed that BDNF/TrkB were co-expressed in the neurons of the TG, and that BDNF and GFAP showed less co-localization in the TG, which suggested that the neurons might have released BDNF through autocrine secretion. Indeed, it has been reported that BDNF is released by TG’s neurons through autocrine activity^10,29^. However, the autocrine secretion of BDNF in TG requires further research to obtain more direct evidence.

Palmatine is an isoquinoline alkaloid extracted from the dried rhizome of *Radix stephaniae*^30^. It has been used clinically to treat bacterial and viral infections in China for thousands of years^31,32^. In our lab, we have found that palmatine relieved hyperalgesia and depression of diabetic rats^21^. Currently on PubMed, however, there are no studies on the therapeutic effect of palmatine on TN. Our research shows that palmatine significantly alleviates the mechanical allodynia of the TN group, suggesting that palmatine may be a potential drug to treat TN. In the TN + palmatine group, the expression of BDNF and TrkB receptor had decreased significantly compared to the TN group. Therefore, we speculate that the effects of palmatine may be related to blockage of the BDNF/TrkB pathway. Between the sham group and sham + palmatine group, the results showed no differences, which demonstrated that palmatine dose used was safe and had few adverse reactions in this experiment.

Some findings suggest that there is a TrkB-ERK/Akt pathway and BDNF-ERK signalling in the cell, and that the activation of the BDNF/TrkB pathway may increase the levels of ERK1/2 and p-ERK1/2^33–35^. Our study also shows that the expression of p-ERK1/2 in the TG of TN rats was remarkably higher than that in the sham rat, but palmatine administration could reduce this expression to comparable levels. These results suggest that palmatine can exert an analgesic effect on TN, potentially through the downregulation of the ERK1/2 pathway. Thus, it is tempting to speculate that the role of ERK phosphorylation in the TG is related to the BDNF/TrkB-mediated TN pain transmission, and that palmatine administration may affect the phosphorylation of ERK in TN rats. There are multiple pathways in pain transmission besides ERK1/2 pathway, therefore, there might be other involved pathways which need further research in the future.

## Conclusion

Our experiments have demonstrated that palmatine administration increases the mechanical pain threshold of TN rats by reducing the expressions of BDNF and the TrkB receptor and by inhibiting the ERK1/2 pathway in TG. These findings suggest that palmatine and its analgesic mechanism deserve further study as a potential pharmacotherapy in the treatment of TN and other chronic pain conditions.

## Materials and Methods

### TN pain animal model

Healthy Sprague-Dawley rats (male, 180–220 g) were obtained from the Department of Animal Science, Nanchang University of Traditional Chinese Medicine. The procedures were approved by the Animal Care and Use Committee of Nanchang University Medical School. The animals had free access to food and water in a room with a comfortable temperature of 20 °C to 25 °C. The International Association for the Study of Pain (IASP)’s ethical guidelines for pain research in animals were followed^36^.

These rats were randomly divided into 4 groups: sham, TN, sham + palmatine, and TN + palmatine. For the TN group, the right infraorbital nerve was exposed and loosely ligated through a previously described method, but for the sham group, the right infraorbital nerve was exposed but not ligated^36^. For the sham + palmatine group and the TN + palmatine group, 10% palmatine was administered through an intraperitoneal injection of 20 mg/kg a day for 2 weeks.

### Evaluation of mechanical allodynia

Mechanical allodynia was measured as described in detail previously in our published paper^36^. The mechanical nociceptive threshold was evaluated using electronic pain meter (BME-404 NO.E5489, Institute of Biomedical Engineering, CAMS & PUMC). The stimulation site was the facial area overlying the infraorbital nerve, i.e., the whisker portion in the centre of the nasal region and the surrounding hairy skin. The threshold of mechanical stimulation intensity of the right side of the rat’s face was measured 1, 3, 5, 7, 9, 11, and 13 days after the operation. Each rat was stimulated 6 times at 1-minute intervals. The electronic mechanical pain tester transmitted the stimulation pressure to the computer’s corresponding software (BME-Tactile), which automatically calculated the mechanical pain threshold of the camp. Animal positive reaction performance included: (1) rapid retreat, dodging and turning, or rats to avoid facial irritation, curling up to the cage wall, or hiding the head and face under the body; (2) aggressive behaviours by biting; (3) scratching the face and repetitive scratching in the same location for at least 3 consecutive times. Any 1 or more of the above 3 reactions were considered to be positive for the stimulation test, i.e., the stimulation threshold was an effective threshold.

### Quantitative real-time PCR

Total RNA was isolated from the right trigeminal ganglion using the TRIzol Total RNA Reagent (TransGen Biotech Company). Reverse transcription to cDNA was performed according to the kit instructions (EasyScript One-Step gDNA Removal and cDNA Synthesis SuperMix, TransGen Biotech). The primers were designed with Primer Express 3.0 Software (Applied Biosystems) and the sequences were as follows: BDNF: Forward: 5′-CCTCTGCTCTTTCTGCTGGA-3′, Reverse: 5′-GCTGTGACCCACTCGCTAAT-3′;TrkB: Forward: 5′-TGGAGGAAGGGAAGTCTGTG-3′, Reverse: 5′-AGTGGTGGTCTGAGGTTGGA-3′; β-actin: Forward: 5′-AAGATCCTGACCGAGCGTGG-3′, Reverse: 5′-CAGCACTGTGTTGGCATAGAGG-3′. The quantification of gene expression was obtained using the ABI PRISM ® 7500 Sequence Detection System (Applied Biosystems Inc., Foster City, USA).

### Immunohistochemistry

On the 14th day, the rats were anesthetized with 10% chloral hydrate (Shanghai Macklin Biochemical Co., Ltd). We then cut the skin and the diaphragm under the costal margin of the rat, exposed the heart, flushed the blood with 0.9% saline, and infused 500 ml of 4% paraformaldehyde (PFA) until the body became stiff. Next, the rat was decapitated, the right trigeminal ganglion was removed and fixed in 4% PFA for 24 hours before being dehydrated for 24 hours. Next, the TG was embedded and cut into 10-μm-thick sections, naturally dried, and stored in a −20 °C freezer. The slices were then incubated in 3% H_2_O_2_ for 10 minutes at 37 °C. To block endogenous peroxidase activity and nonspecific antigen binding, the processed slices were incubated with 10% goat serum for 30 minutes at 37 °C. The sections were then washed in PBS and incubated with rabbit anti-BDNF or anti-TrkB (BDNF 1:50 diluted in PBS; Abcam company; TrkB 1:100, Abcam company) overnight at 4 °C. After washing 3 times with PBS, the sections were incubated with biotinylated goat anti-rabbit secondary antibodies (Beijing Zhongshan Biotech Co., China) for 30 minutes at 37 °C. After washing with PBS, the samples were stained with DAB kit (Beijing Zhongshan Biotech Co., China) for 3–5 minutes. Immunohistochemical results were obtained by inverted fluorescence microscope (Olympus, Tokyo, Japan). After immunohistochemistry, Image-Pro Plus 6.0 software was used to analyse the changes in the levels of BDNF/TrkB determined with integrated optical density (IOD) in the ganglia. The background level was determined by averaging the optical density of 10 random areas^21^.

### Immunofluorescence

Immunofluorescence was then studied. The TG iced-slices were washed in PBS and incubated in a blocking solution containing 3% bovine serum albumin (BSA) in PBS with 0.3% Triton X-100 (Solarbio Company, Beijing, China) for 30 minutes at room temperature. In 4 °C refrigerator primary antibodies (mouse anti-GFAP, Abcam Company, 1:200; rabbit anti-BDNF, Abcam, 1:500) were incubated overnight. The samples were washed using PBS and the secondary antibody (goat anti-rabbit TRITC, Jackson ImmunoResearch Inc., West Grove, PA, USA, 1:200; goat anti-mouse FITC, Beijing Zhongshan Biotech Co., 1:200) was added for 45 minutes at 37 °C. Controls omitted the primary antibody. A fluorescence microscope (Olympus, Japan) and Image-Pro Plus 6.0 software were used to detect the immunofluorescence intensity of BDNF or GFAP expression. The co-expression steps of TrkB and GFAP were the same as mentioned above, except for the different dilution ratio (TrkB 1:1000, GFAP 1:200)^27^.

### Western blotting experiment

After anaesthetizing the rats with 10% chloral hydrate (Shanghai Xinya Medical Company, Shanghai, China), the TG tissues were taken out and homogenized with a detergent lysis buffer containing phenylmethanesulfonyl fluoride (PMSF). The lysate was then transferred to a 1.5 ml centrifuge tube with a pipette and centrifuged at 12000 rpm for 5 minutes at 4 °C. The supernatant was collected and placed at −20 °C. The total protein in the supernatant was measured using the Lowry method. The protein was diluted with a sample buffer and at 95 °C for 10 minutes. SDS-polyacrylamide gel (12%) electrophoresis was used to separate the same amount of proteins (20 μg), and then transferred onto a nitrocellulose (NC) membrane in a Bio-Rad electrophoresis device. Primary antibodies (rabbit polyclonal anti-BDNF, Abcam; TrkB, Abcam; ERK1/2 or p- ERK1/2, Cell Signaling Technology; and β-actin, Advanced Immunochemicals) were used. The secondary antibody, goat anti-rabbit IgG, was purchased from Beijing Zhongshan Biotech. Using a Bio-Rad system, the labelled proteins were then imaged with an enhanced chemiluminescence. The optical density of the target proteins band were calculated using Image-Pro Plus software after normalizing to each β-actin band^36^.

### Statistical analysis

The data were analysed using the SPSS software (version 21.0). All results are represented as the mean ± SEM. Statistical significance was analysed with a one-way analysis of variance (ANOVA), and was followed, when appropriate, by Fisher’s post hoc test for multiple comparisons. Results were considered significant when p < 0.05.

### Ethical approval

The Animal Care and Ethics Committee of Nanchang University approved the procedures of this research. The IASP’s ethical guidelines for the study of pain in animals were followed.

## Supplementary information

Supplementary information.

## Data Availability

The datasets generated during and/or analyzed during the current study are available from the corresponding author upon reasonable request.
