# Supplementary material for: Effects of palmatine on BDNF/TrkB-mediated trigeminal neuralgia
Source: Sci Rep. 2020 Mar 19;10:4998. doi: 10.1038/s41598-020-61969-1 (PMC7081188; doi:10.1038/s41598-020-61969-1)
Supplement: Supplementary file 1 — Supplementary information. [file 41598_2020_61969_MOESM1_ESM.docx]

**Supplementary information**

For the gels and blots, we only selected a portion of the results of the Western blot that represents this experiment. And in the relevant experimental research, the expression of this result is also adopted.

Original immunoblots are provided as follows.


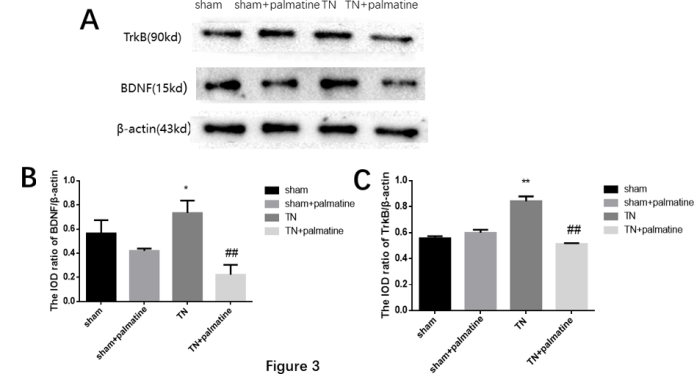


The original full-length gel of TrkB of figure3:


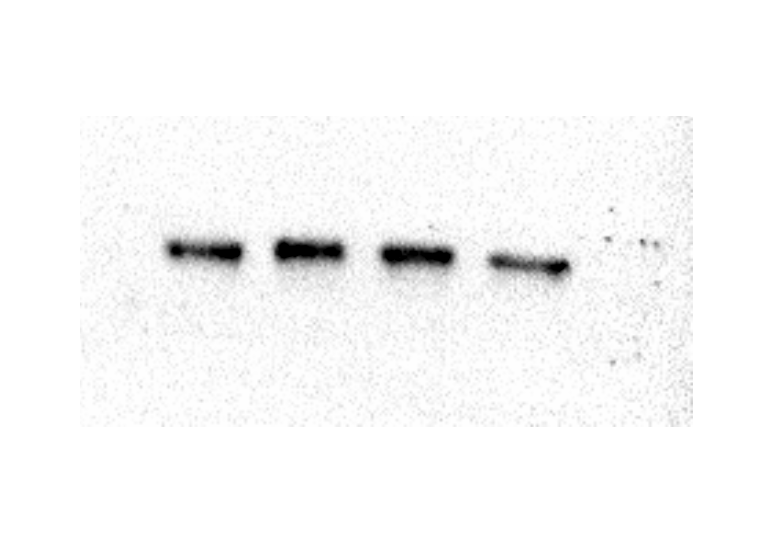


The original full-length gel of BDNF of figure3:


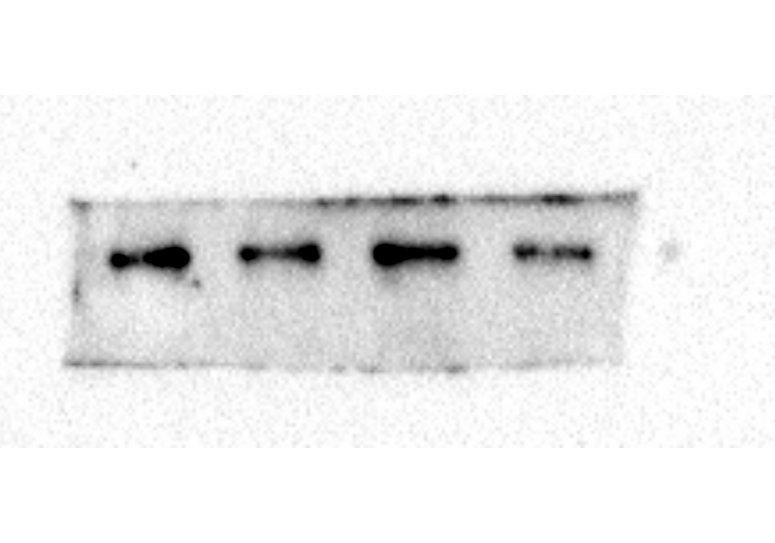


The original full-length gels of β-actin of figure3:


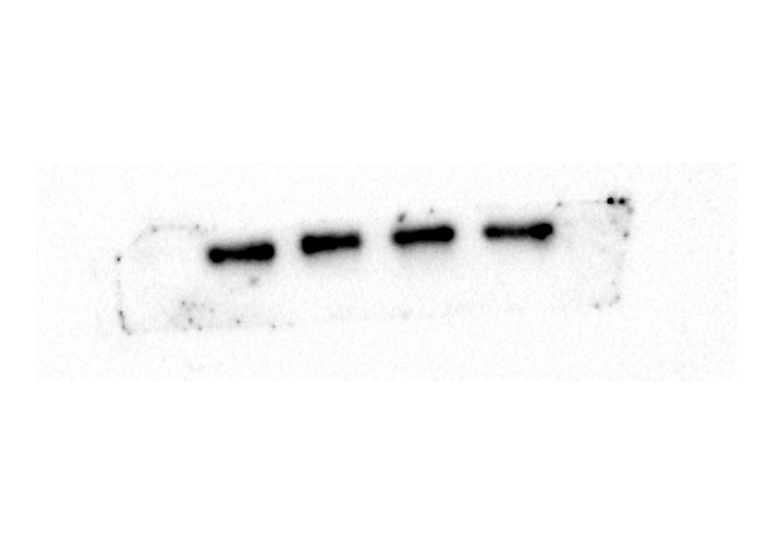


The three original full-length gels were come from the same gel.


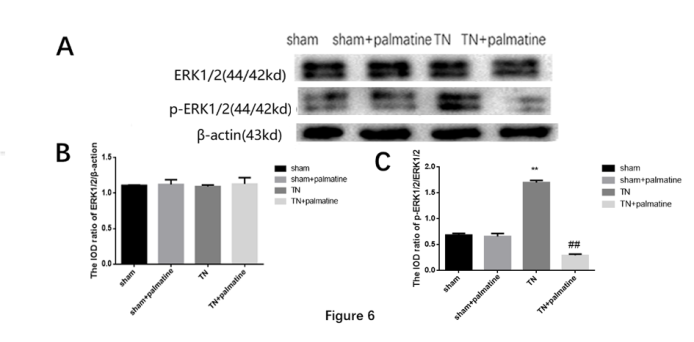


The original full-length gel of ERK1/2 of figure6:


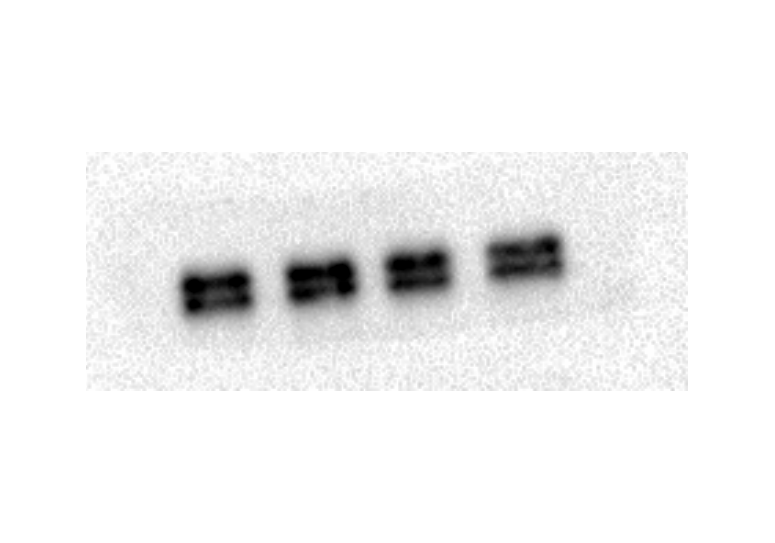


The original full-length gel of p-ERK1/2 of figure6:


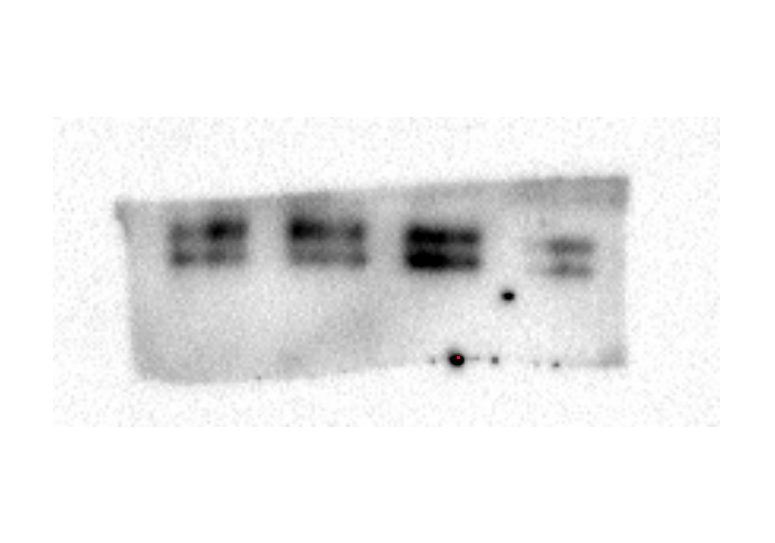


The original full-length gels of β-actin A of figure6:


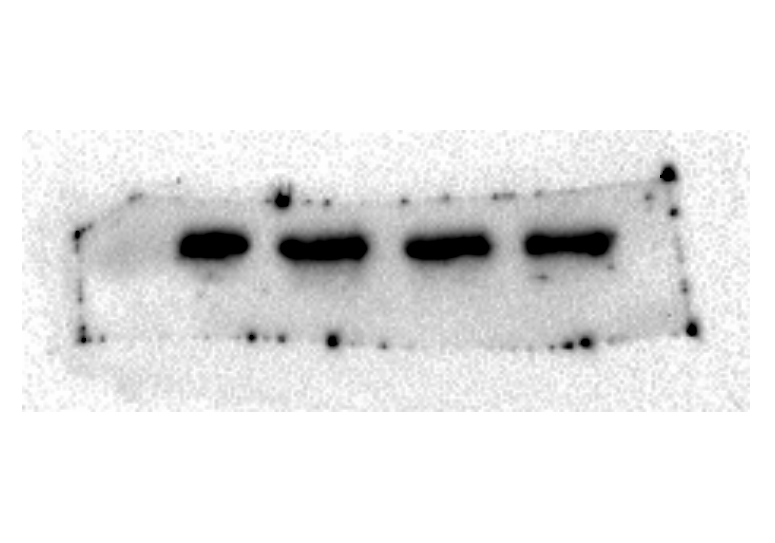


The three original full-length gels were come from different gels using the equal quantity protein.

**Title page**

**1.Title:** Effects of palmatine on BDNF/TrkB-mediated trigeminal neuralgia

**2. Running Title:** Palmatine alleviates trigeminal neuralgia by inhibiting BDNF/TrkB

**3. All authors and institution:**

Lijuan Liu^1,2,a^, Lingkun He^1,a^, Cancan Yin^1,a^, Ruoyu Huang ^1,a^, Wenhao Shen ^1^, Huixiang Ge^3^, Mengyun Sun^3^, Shujuan Li^4^ , Yun Gao^3^, Wei Xiong^1, 5*^

^1^ Affiliated Stomatological Hospital of Nanchang University, Nanchang, Jiangxi, China

^2^ First Affiliated Hospital of Nanchang University, Nanchang, Jiangxi, China

^3^ Department of Physiology, Basic Medical College, Nanchang University, Nanchang, Jiangxi, China

^4^ Second Clinic Medical College of Grade 2017, Nanchang University, Nanchang, Jiangxi, China

^5^Jiangxi Provincial Key Laboratory of Oral Biomedicine, Nanchang, Jiangxi, China

**4. Corresponding author:** Dr. Wei Xiong

Affiliated Stomatological Hospital of Nanchang University

39 Fuzhou Road, Nanchang 330006, Jiangxi, P. R. China

Tel: 0086-791-863606156;

Fax: 0086-791-86360615;

E-mail: xiongwei96@163.com

^a^These four authors contributed equally to this work.
